# Nomogram model of mortality risk in patients with chronic obstructive pulmonary disease in intensive care unit: based on MIMIC-IV database and external validation study

**DOI:** 10.3389/fmed.2025.1547047

**Published:** 2025-07-22

**Authors:** Yikun Guo, Chen Zuo, Jun Yan, Chengjun Ban

**Affiliations:** ^1^Dongzhimen Hospital, Beijing University of Chinese Medicine, Beijing, China; ^2^Beijing University of Chinese Medicine, Beijing, China

**Keywords:** MIMIC-IV, chronic obstructive pulmonary disease, mortality risk, nomogram, external validation, intensive care unit

## Abstract

**Objective:**

Chronic obstructive pulmonary disease (COPD) is a common respiratory disease with high incidence and mortality rates. This study aims to identify independent risk factors affecting the mortality risk of COPD patients and construct and validate a nomogram model to provide treatment guidance for COPD patients.

**Methods:**

Data from COPD patients in the intensive care unit (ICU) were obtained from the Medical Information Mart for Intensive Care-IV (MIMIC-IV) and the eICU Collaborative Research Database (eICU-CRD). The MIMIC-IV dataset was randomly divided into training and testing sets in a 7:3 ratio for model development and evaluation. External validation was performed using the eICU-CRD dataset. Independent prognostic factors were determined using multivariable Cox regression analysis and incorporated into the nomogram. The performance and clinical applicability of the prediction model were evaluated using the concordance index, receiver operating characteristic (ROC) curve, calibration curve, and decision curve analysis (DCA).

**Results:**

The MIMIC-IV dataset included 2036 COPD patients, and the eICU-CRD dataset included 13,053 COPD patients. The constructed nomogram model included 7 variables: age, weight, APSIII score, ventilation duration, potassium ion, anion gap, and international normalized ratio. Among these factors, ventilator time was a protective factor, while the remaining six factors were independent risk factors. The nomogram demonstrated good accuracy with C-index values of 0.862, 0.874, and 0.722 in the training set, testing set, and external validation set, respectively. The ROC curve indicated good predictive performance of the nomogram model, and the calibration curve and DCA further confirmed the reliability and clinical utility.

**Conclusion:**

This study established a simple and effective nomogram model consisting of 7 variables for evaluating the short-term mortality risk of COPD patients. It provides better recommendations for clinical decision-making and improves the short-term survival rate of COPD patients.

## 1 Introduction

Chronic Obstructive Pulmonary Disease (COPD) is a leading cause of global mortality and disability with large economic burden (1, 2). COPD is a heterogeneous lung disease characterized by chronic airway inflammation, lung tissue damage, and persistent, progressive airflow obstruction (3–5). Due to prolonged exposure to risk factors and changes in the global population age structure, the medical and economic burdens of COPD are expected to surge, with over 200 million COPD patients worldwide in 2019, and projected to become the third leading cause of death by 2030 (6).

Nomogram models have been widely used as predictive tools for various diseases, integrating independent prognostic factors to construct predictive models for assessing patient survival probabilities, and are commonly used in medical research and clinical practice (7). Studies have shown that current nomogram models for COPD primarily assess ICU hospitalization duration, lung function, and disease exacerbations in COPD patients (8–10). Sakamoto et al. (11) collected hospitalization data of COPD patients in Japan to construct a prognostic nomogram, but the included variables were limited, and internal and external validation was not conducted, resulting in limitations in the predictive results of the column chart. Therefore, it is crucial to develop a rapid and accurate column chart model (nomogram) for predicting the mortality risk of COPD patients.

This study conducted a retrospective cohort study using the Critical Care Medical Information Market IV (MIMIC-IV) database, and screened independent prognostic factors for COPD patients through multivariate COX regression analysis. A column chart model was constructed to predict the short-term mortality risk of COPD patients. In addition, we further collected relevant clinical data of COPD patients in the eICU Collaborative Research Database (eICU-CRD), applied the newly developed predictive model to the dataset, and then conducted external validation to evaluate its clinical applicability and value.

This study constructed a nomogram for predicting the short-term mortality risk of COPD patients using clinical data from the MIMIC-IV database, and conducted rigorous internal and external validation, which helps clinicians evaluate the mortality risk of COPD patients and develop personalized treatment strategies.

## 2 Materials and methods

### 2.1 Data source

The clinical data of COPD patients included in this study were obtained from the MIMIC-IV database (version 2.2) and eICU-CRD (version 2.0). MIMIC-IV is a large, freely accessible database that includes over 50,000 ICU admissions from Beth Israel Deaconess Medical Center (BIDMC) between 2008 and 2019 (12). The eICU-CRD is a national telemedicine database, containing information from over 200,000 patients admitted to 335 ICUs in 208 hospitals across the United States between 2014 and 2015 (13). Both databases provide extensive clinical data, including demographics, vital signs, laboratory results, medication use, and nursing records. This study involved secondary analysis of publicly available databases, MIMIC-IV and eICU-CRD, therefore, no ethical approval was required. Moreover, as all patient information in the databases has been deidentified, informed consent was not necessary. The first author (Yikun Guo) has completed a training program provided by the collaborating institution and obtained a certificate (number: 62099487) qualifying for access to the databases and retrieval of information.

### 2.2 Study population

**Figure 1:**
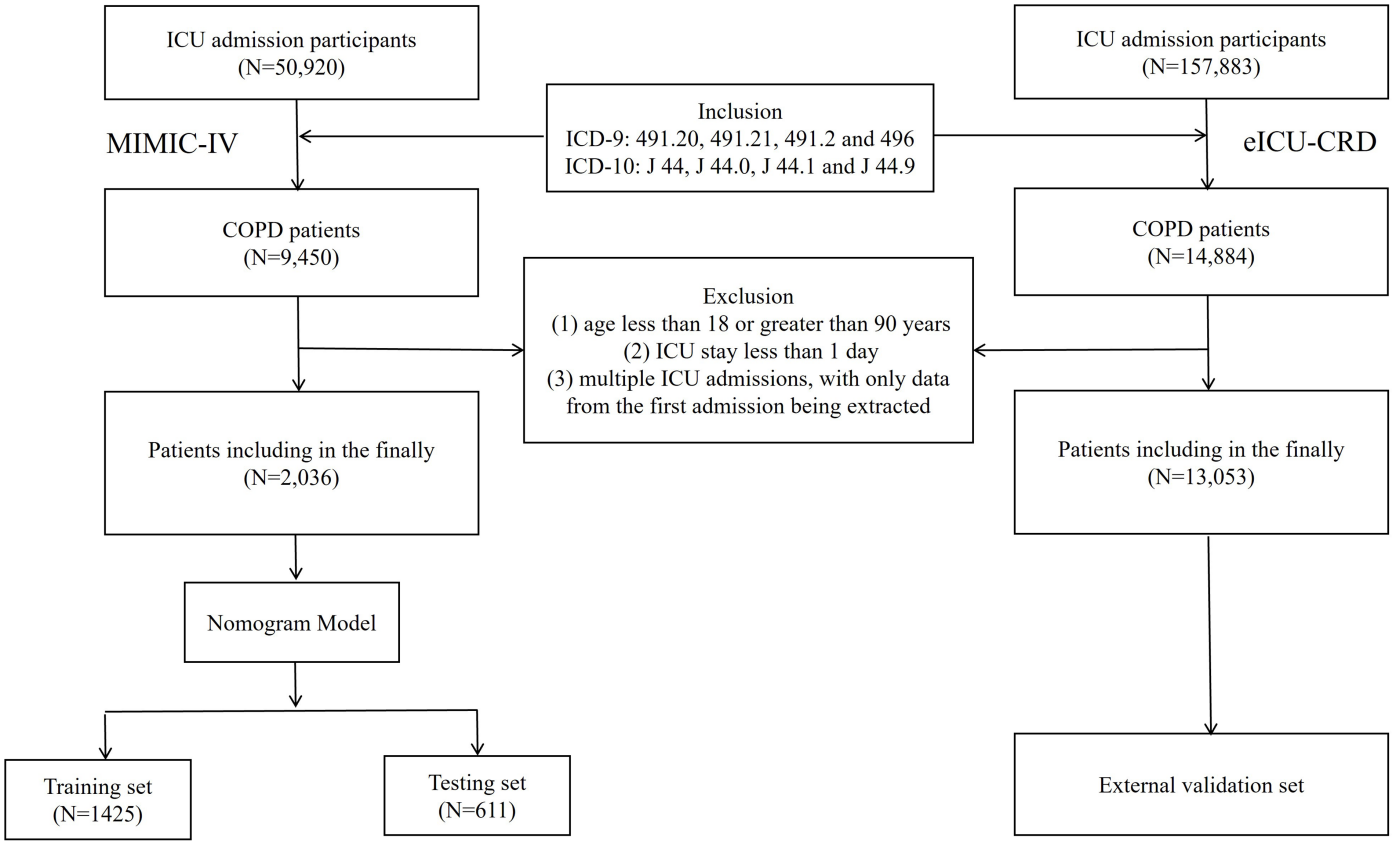
Selecting flowchart.

This study included patients diagnosed with COPD based on ICD-9 and ICD-10 codes. The following inclusion require was applied: patients were diagnosed with CODP by the International Classification of Disease. (ICD-9 codes: 491.20, 491.21, 491.2 and 496; ICD-10 codes: J 44, J 44.0, J 44.1 and J 44.9). The following exclusion criteria were applied: (1) age less than 18 or greater than 90 years, (2) ICU stay less than 1 day, and (3) multiple ICU admissions, with only data from the first admission being extracted. Ultimately, a total of 15,089 patients were included in this retrospective study, with 2,036 patients from the MIMIC-IV database and 13,053 patients from the eICU-CRD database. Patients from the MIMIC-IV dataset were randomly allocated to a training set and testing set in a 7:3 ratio, while the patients from the eICU-CRD dataset constituted the external validation set (Figure 1).

### 2.3 Data extraction

Utilizing PostgreSQL software (version 13.7.2) and PgAdmin4, relevant clinical data readily accessible from the MIMIC-IV database and eICU-CRD database were extracted to ensure the practicality of the study. All data in this study were extracted using Structured Query Language (SQL). The extracted data encompassed six categories: (1) Demographics: age, gender, and weight. (2) Vital signs: heart rate (HR), mean blood pressure (MBP), systolic blood pressure (SBP), diastolic blood pressure (DBP), respiratory rate (RR), temperature, and oxygen saturation (SpO2). (3) Laboratory indicators: white blood cell count (WBC), red blood cell count (RBC), platelet count (PLT), hemoglobin (HGB), chloride (Cl), potassium (K), sodium (Na), calcium (Ca), magnesium (Mg), anion gap (AG), creatinine (Cr), blood urea nitrogen (BUN), international normalized ratio (INR), activated partial thromboplastin time (APTT), pH, partial pressure of carbon dioxide (PCO2), and partial pressure of oxygen (PO2). (4) Complications: systemic inflammatory response syndrome (SIRS), hypertension, diabetes, heart failure (HF), myocardial infarction, cancer, pneumonia, stroke, atrial fibrillation, kidney failure, and coronary heart disease. (5) Scoring systems: sequential organ failure assessment (SOFA) score, simplified acute physiology score II (SAPSII), Acute Physiology Score III (APS III), Charlson comorbidity index (CCI). (6) Treatment information included mechanical ventilation, mechanical ventilation duration, and the use of antibiotic. (7) Outcome indicators: ICU survival status, ICU length of stay (LOS).

### 2.4 Statistical analysis

We used the Kolmogorov–Smirnov test to assess the normality of continuous variables. Normally distributed continuous variables were described as mean ± standard deviation and compared between groups using the t-test. Non-normally distributed continuous variables were expressed as interquartile ranges (IQR) and compared between groups using the Wilcoxon rank-sum test. Categorical variables were presented as numbers and percentages, and differences between groups were compared using the chi-square test or Fisher’s exact probability.

This study employed multivariable Cox regression analysis to identify independent prognostic factors in COPD patients. The variance inflation factor (VIF) was calculated to assess the collinearity between variables. Based on the selected variables, a nomogram model for short-term prognosis in COPD patients was developed. The performance of the model was further evaluated using a testing set, with the C-index and receiver operating characteristic (ROC) curve used to assess the predictive ability of the nomogram. The accuracy of the model was assessed using calibration curves. Decision curve analysis (DCA) was performed to evaluate the clinical utility of the nomogram. External validation was conducted using the eICU-CRD dataset.

To ensure the reliability of the analysis, variables with more than 20% missing values in vital signs and laboratory indicators were excluded, and other missing variables were handled using imputation. Each statistical test was conducted with a two-tailed design. R version 4.2.2 was used for statistical analysis, and *p*-values≤0.05 were considered statistically significant.

## 3 Results

A total of 2,036 participants were recruited from the MIMIC-IV database based on inclusion and exclusion criteria, including 1,089 (53.5%) males and 947 (46.5%) females, with an average age of 71.8 years. During the ICU stay, 186 patients died, resulting in an ICU mortality rate of 9.13% and an average ICU stay of 4.8 days.

### 3.1 Baseline characteristics

Table 1 presents the clinical characteristics and baseline data of the participants. The 2,036 participants were randomly allocated to training and testing sets at a 7:3 ratio, with 1,425 in the training set and 611 in the testing set, over half of whom were older adult males. Regarding comorbidities, only 0.8% of patients did not have systemic inflammatory response syndrome (SIRS), and over one-third of patients had cardiovascular and endocrine diseases, such as heart failure (43%), coronary heart disease (39.7%), hypertension (37.9%), and diabetes (33.8%); the prevalence of cancer (19.7%) and stroke (8.9%) as comorbidities was lower. Based on the four included scoring systems, COPD patients likely had sepsis and unstable physiological conditions, indicating a high risk of death. Laboratory indicators suggested possible infections and anemia in the patients. In terms of treatment, most patients received antibiotics (80.5%) and mechanical ventilation (86.3%), with an average duration of mechanical ventilation of 75.9 h. There were no significant baseline characteristic differences between the training set (*N* = 1,425) and the testing set (*N* = 611), indicating that the two groups were comparable (*p* > 0.05).

**Table 1:** Baseline characteristics of COPD patients.

Characteristics	Training set (*N* = 1,425)	Testing set (*N* = 611)	Total (*N* = 2036)	*P*-value
Demography				
Gender, n (%)				0.870
Female	665 (46.7)	282 (46.2)	947 (46.5)	
Male	760 (53.3)	329 (53.8)	1,089 (53.5)	
Age (year)	71.8 [60.9, 82.7]	71.7 [60.5, 82.9]	71.8 [60.8, 82.8]	0.857
Weight (kg)	82.4 [57.7, 107.1]	81.5 [57, 106]	82.1 [57.4, 106.8]	0.469
Vital signs				
HR (times/min)	89.1 [68.9, 109.3]	88.1 [68.8, 107.4]	88.8 [68.8, 108.8]	0.292
RR (times/min)	20.1 [13.5, 26.7]	19.9 [13.2, 26.6]	20 [13.4, 26.6]	0.485
Temperature (°C)	36.8 [35.8, 37.8]	36.8 [36.2, 37.4]	36.8 [35.9, 37.7]	0.854
SBP (mmHg)	121 [96.3, 145.7]	121 [96.7, 145.3]	121 [96.4, 145.6]	0.998
DBP (mmHg)	69 [50.7, 87.3]	69.3 [50.2, 88.4]	69.1 [50.5, 87.7]	0.792
MBP (mmHg)	82.7 [64, 101.4]	92.4 [63.4, 100.8]	85.6 [63.8, 101.2]	0.327
SpO_2_ (%)	96.2 [91.7, 100.7]	96.4 [92.8, 100]	96.2 [92, 100.4]	0.282
Comorbidities, n (%)				
SIRS				0.978
0	11 (0.8)	5 (0.8)	16 (0.8)	
1	174 (12.2)	69 (11.3)	243 (11.9)	
2	482 (33.8)	211 (34.5)	693 (34)	
3	603 (42.3)	257 (42.1)	860 (42.2)	
4	155 (10.9)	69 (11.3)	224 (11)	
Hypertension				0.935
No	886 (62.2)	378 (61.9)	1,264 (62.1)	
Yes	539 (37.8)	233 (38.1)	772 (37.9)	
Diabetes mellitus				0.916
No	945 (66.3)	403 (66)	1,348 (66.2)	
Yes	480 (33.7)	208 (34)	688 (33.8)	
Heart failure				0.191
No	798 (56)	362 (59.2)	1,160 (57)	
Yes	627 (44)	249 (40.8)	876 (43)	
Myocardial infarction				0.961
No	1,197 (84)	512 (83.8)	1709 (83.9)	
Yes	228 (16)	99 (16.2)	327 (16.1)	
Cancer				0.701
No	1,148 (80.6)	487 (79.7)	1,635 (80.3)	
Yes	277 (19.4)	124 (20.3)	401 (19.7)	
Pneumonia				0.962
No	967 (67.9)	416 (68.1)	1,383 (67.9)	
Yes	458 (32.1)	195 (31.9)	653 (32.1)	
Stroke				0.757
No	1,296 (90.9)	559 (91.5)	1855 (91.1)	
Yes	129 (9.1)	52 (8.5)	181 (8.9)	
Atrial fibrillation				0.310
No	848 (59.5)	379 (62)	1,227 (60.3)	
Yes	577 (40.5)	232 (38)	809 (39.7)	
Kidney failure				0.327
No	977 (68.6)	433 (70.9)	1,410 (69.3)	
Yes	448 (31.4)	178 (29.1)	626 (30.7)	
Coronary heart disease				0.139
No	844 (59.2)	384 (62.8)	1,228 (60.3)	
Yes	581 (40.8)	227 (37.2)	808 (39.7)	
Scores				
SOFA	5.1 [1.5, 8.7]	5 [1.5, 8.5]	5.1 [1.5, 8.7]	0.488
APS III	45 [25.8, 64.2]	45.1 [25.7, 64.5]	45 [25.8, 64.2]	0.928
SAPS II	38.7 [25.7, 51.7]	38.7 [26.2, 51.2]	38.7 [25.9, 51.5]	0.997
CCI	6.7 [4, 9.4]	6.7 [3.9, 9.5]	6.7 [3.9, 9.5]	0.945
Blood gas				
AG (mmol/L)	14.6 [11, 18.2]	14.5 [10.6, 18.4]	14.6 [10.9,18.3]	0.821
pH	7.4 [7.3, 7.5]	7.4 [7.3,7.5]	7.4 [7.3,7.5]	0.564
PCO_2_ (mmHg)	46.5 [36.4, 56.6]	46.8 [37, 56.6]	46.6 [36.6, 56.6]	0.487
PO_2_ (mmHg)	115.8 [48.3, 183.3]	114 [50.2, 177.8]	115.3 [48.9, 181.7]	0.576
Laboratory tests				
WBC (K/UL)	13 [5.8, 20.2]	13.5 [−2.8, 29.8]	13.1 [2.3, 23.9]	0.465
RBC (m/uL)	3.5 [2.8, 4.2]	3.5 [2.8, 4.2]	3.5 [2.8, 4.2]	0.180
PLT (K/μL)	199.9 [106.2, 293.6]	194.3 [106.4, 282.2]	198.2 [106.2, 290.2]	0.207
HGB (g/dL)	10.4 [8.4, 12.4]	10.3 [8.3, 12.3]	10.4 [8.4, 12.4]	0.061
Na (mmol/L)	138.5 [133.8, 143.2]	138 [133, 143]	138.3 [133.5, 143.1]	0.059
K (mmol/L)	4.3 [3.7, 4.9]	4.4 [3.8, 5]	4.3 [3.7, 4.9]	0.216
Ca (mg/dL)	8.4 [7.7, 9.1]	8.4 [7.8, 9]	8.4 [7.7, 9.1]	0.381
Cl (mmol/L)	102.1 [96.3, 107.9]	101.5 [95.3, 107.7]	101.9 [96, 107.8]	0.069
Mg (mmol/L)	2.1 [1.6, 2.6]	2 [1.5, 2.5]	2 [1.5, 2.5]	0.181
APTT (s)	38.6 [19.5, 57.7]	38.8 [21, 56.6]	38.7 [20, 57.4]	0.872
INR	1.4 [0.8, 2]	1.4 [0.8, 2]	1.4 [0.8, 2]	0.533
BUN (mg/dL)	27.6 [6.4, 48.8]	28.4 [5.8, 51]	27.8 [6.2, 49.4]	0.436
Cr (mg/dL)	1.4 [0.3, 2.5]	1.4 [0, 2.8]	1.4 [0.2, 2.6]	0.724
Treatment				
Mechanical ventilation, n (%)				0.770
No	192 (13.5)	86 (14.1)	278 (13.7)	
Yes	1,233 (86.5)	525 (85.9)	1758 (86.3)	
Mechanical ventilation duration (hours)	75.3 [19.2, 169.8]	77.4 [14.1, 168.9]	75.9 [17.7, 169.5]	0.639
Antibiotic, n (%)				0.595
No	273 (19.2)	124 (20.3)	397 (19.5)	
Yes	1,152 (80.8)	487 (79.7)	1,639 (80.5)	
Outcome				
LOS in ICU (days)	4.8 [1.1, 10.7]	4.8 [0.8, 10.4]	4.8 [1.0, 10.6]	0.848
ICU survival status, n (%)				0.825
Alive	1,293 (90.7)	557 (91.2)	1850 (90.9)	
Dead	132 (9.3)	54 (8.8)	186 (9.1)	

HR: Heart rate; RR: Respiratory rate; DBP: Diastolic blood pressure; SBP: Systolic blood pressure; MBP: Mean blood pressure; SpO_2_: Blood oxygen saturation; SIRS: systemic inflammatory response syndrome; SOFA: Sequential Organ Failure Assessment; APSIII: Acute Physiology Score III; SAPSII: Simplified Acute Physiology Score II; CCI: Charlson comorbidity index; AG: anion gap; PCO_2_: partial pressure of carbon dioxide; PO_2_: partial pressure of oxygen; WBC: white blood cell count; RBC: red blood cell count; PLT: platelet; HGB: hemoglobin; APTT: activated partial thromboplastin time; INR: international normalized ratio; BUN: Blood urea nitrogen; Cr: Creatinine; LOS: length of stay.

### 3.2 COX regression analysis

Univariable and multivariable Cox regression analyses were conducted on the included variables. Table 2 shows that seven variables were identified as independent prognostic factors in COPD patients. These include age (HR = 1.05, 95%CI: 1.02–1.07, *p* < 0.001), weight (HR = 1.01, 95%CI: 1.00–1.02, *p* = 0.036), APSIII (HR = 1.03, 95%CI: 1.02–1.05, *p* < 0.001), potassium (HR = 1.45, 95%CI: 1.01–2.09, *p* = 0.045), AG (HR = 1.09, 95%CI: 1.01–1.18, *p* = 0.021), INR (HR = 1.20, 95%CI: 1.00–1.44, *p* = 0.048) as independent risk factors for patient prognosis, and ventilator time (HR = 0.99, 95%CI: 0.99–0.99, p < 0.001) as an independent protective factor. Additionally, the VIF values for these variables were all below 4 (age: 1.2852, weight: 1.2148, APSIII: 3.4378, ventilation time: 1.1042, potassium: 1.194, AG: 1.5036, INR: 1.2485), indicating no multicollinearity.

**Table 2:** Univariate and multivariate COX regression analysis.

Characteristics	Univariable analysis			Multivariable analysis		
	HR	95%CI	*p*-value	HR	95%CI	*p*-value
Demography						
Age (year)	1.03	1.01–1.04	0.002	1.05	1.02–1.07	<0.001
Weight (kg)	1.01	1.00–1.02	0.045	1.01	1.00–1.02	0.036
Vital signs						
HR (times/min)	1.01	1.00–1.02	0.031	0.99	0.98–1.00	0.220
SpO_2_ (%)	0.95	0.93–0.98	<0.001	0.97	0.94–1.01	0.146
Comorbidities						
Hypertension						
No (Reference)						
Yes	0.67	0.46–0.98	0.037	0.99	0.59–1.66	0.968
Scores						
SOFA	1.15	1.11–1.20	<0.001	1.04	0.96–1.14	0.339
APS III	1.02	1.02–1.03	<0.001	1.03	1.02–1.05	<0.001
SAPS II	1.04	1.03–1.05	<0.001	0.98	0.96–1.01	0.228
CCI	1.13	1.07–1.20	<0.001	1.06	0.96–1.17	0.254
Blood gas						
AG (mmol/L)	1.12	1.08–1.17	<0.001	1.09	1.01–1.18	0.021
pH	0.00	0.00–0.01	<0.001	0.70	0.01–47.12	0.866
Laboratory tests						
RBC (m/uL)	0.69	0.54–0.89	0.005	1.26	0.69–2.29	0.453
HGB (g/dL)	0.89	0.81–0.97	0.007	0.84	0.67–1.04	0.109
K (mmol/L)	1.63	1.23–2.15	<0.001	1.45	1.01–2.09	0.045
Cl (mmol/L)	0.74	0.57–0.95	0.017	0.88	0.66–1.17	0.370
APTT (s)	1.01	1.00–1.02	0.006	1.01	1.00–1.02	0.075
INR	1.29	1.16–1.44	<0.001	1.20	1.00–1.44	0.048
BUN (mg/dL)	1.01	1.01–1.02	<0.001	0.99	0.97–1.03	0.208
Cr (mg/dL)	1.27	1.15–1.40	<0.001	1.01	0.81–1.26	0.955
Treatment						
Mechanical ventilation						
No (Reference)						
Yes	0.39	0.23–0.65	<0.001	0.61	0.33–1.14	0.123
Mechanical ventilation time(hours)	0.99	0.99–0.99	<0.001	0.99	0.99–0.99	<0.001

### 3.3 Construction and validation of nomogram model

**Figure 2:**
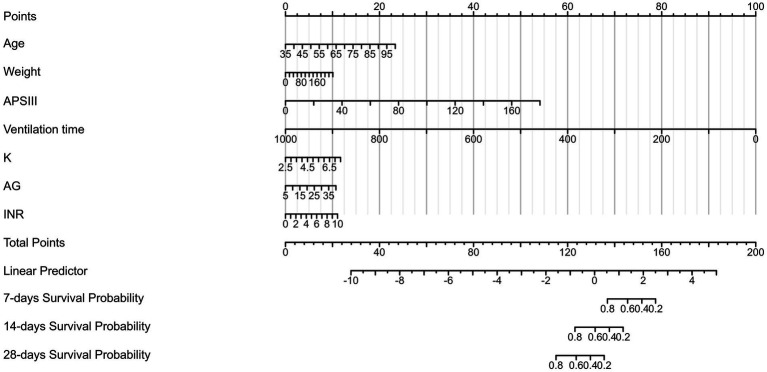
Nomogram for short-term survival probability in COPD patients.

Based on the seven risk factors identified from the COX regression analysis, we developed a nomogram to predict the 7-day, 14-day, and 28-day survival probabilities of COPD patients (Figure 2). The total score for each patient was calculated by summing the points corresponding to each variable, with the total score value representing the predicted probability of prognosis for COPD patients. The C-index in the training and testing sets were 0.862 (0.847–0.877) and 0.874 (0.845–0.904), respectively, indicating good model accuracy.

**Figure 3:**
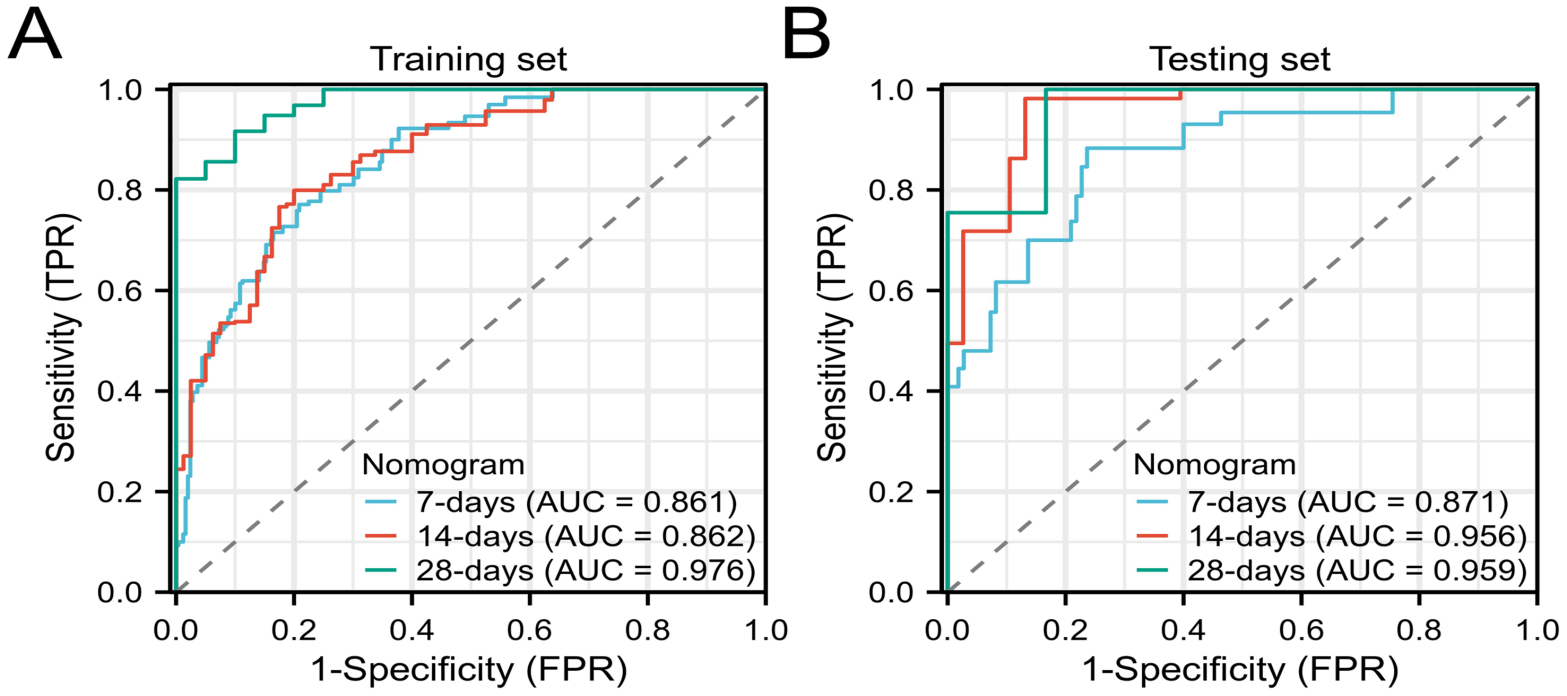
ROC curves of the nomogram model **(A)** Training set; **(B)** Testing set.

**Figure 4:**
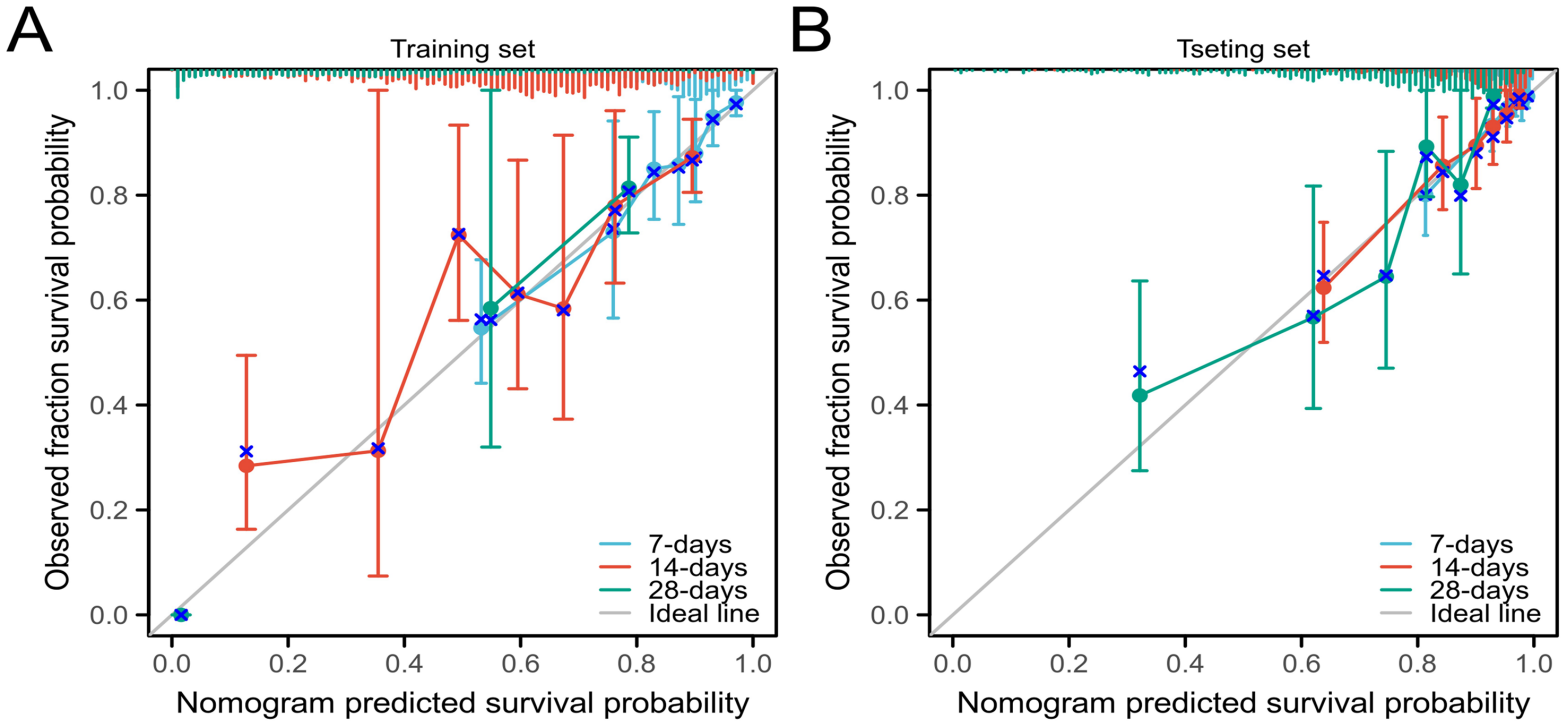
Calibration curves of the nomogram model **(A)** Training set; **(B)** Testing set.

**Figure 5:**
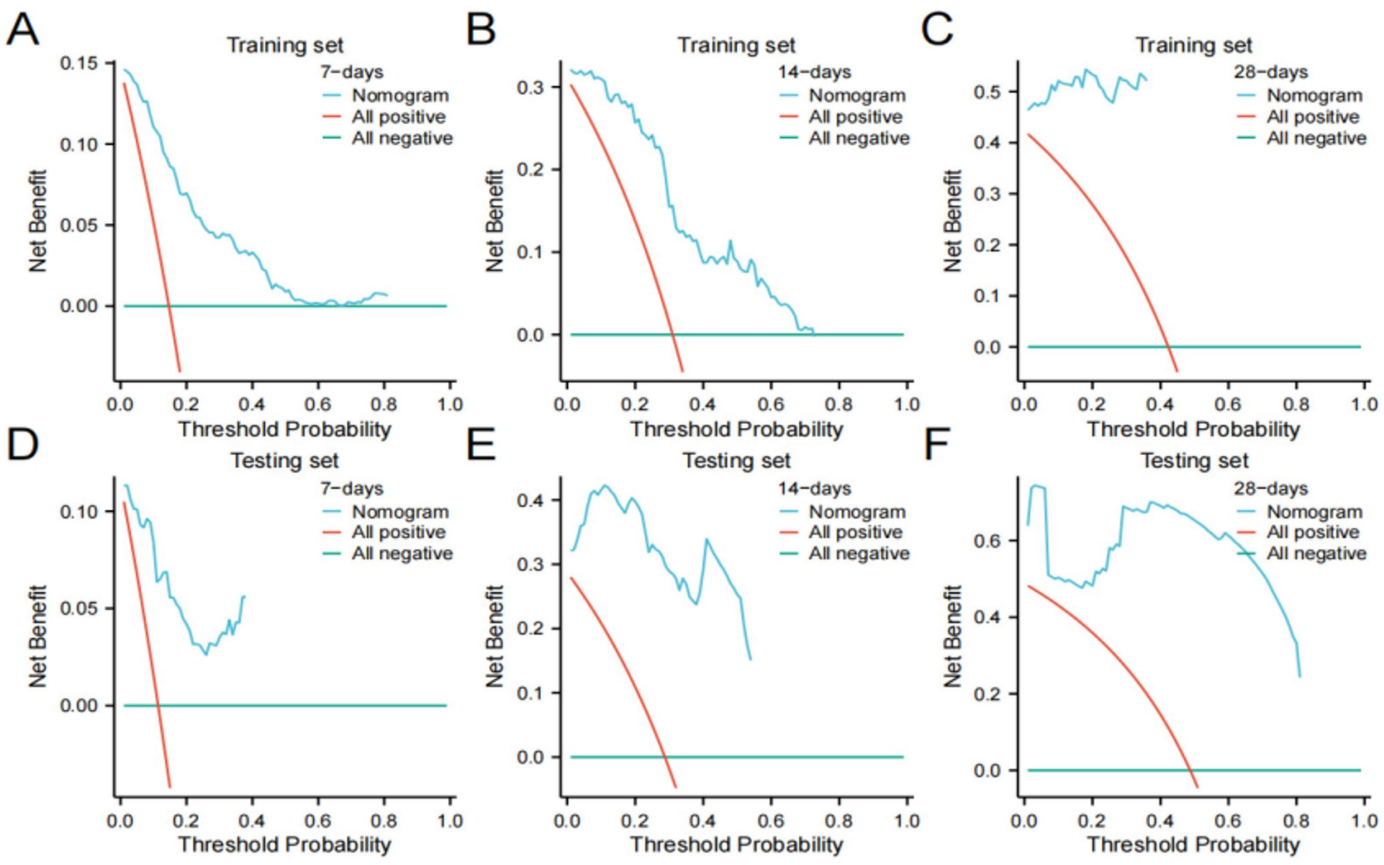
Decision curve analysis (DCA) plots **(A–C)** Training set; **(D-F)** Testing set.

We further validated the accuracy of the nomogram model through ROC curves, calibration curves, and DCA. The ROC curve analysis of the nomogram (Figures 3A,B) showed that the area under the curve (AUC) for 7-day, 14-day, and 28-day mortality risks in the training set were 0.861, 0.862, and 0.976, respectively, and in the testing set were 0.871, 0.956, and 0.959, respectively, indicating good predictive performance of the nomogram. Calibration curves, which more accurately reflect whether the actual outcomes of each nomogram match the predicted outcomes, showed (Figure 4) that the nomograms in both cohorts closely aligned with the diagonal, indicating good fit and consistency with actual prognosis outcomes. DCA evaluates the clinical value of the model by comparing the standardized net benefit and risk threshold probability (14). The green horizontal line represents the benefit when no patients receive intervention, the diagonal red line represents the benefit when all patients receive intervention, and the blue curve represents the benefit when patients receive intervention based on the model’s judgment. The DCA curve (Figure 5) results showed that clinical interventions guided by the nomogram model yielded greater net benefits in both the training and testing sets.

### 3.4 External validation

In addition to using the MIMIC-IV dataset as the training and internal testing sets, data from the eICU-CRD database were collected as the external validation set. The external validation set included 13,053 COPD patients and was used to independently validate the newly developed prediction model.

**Figure 6:**
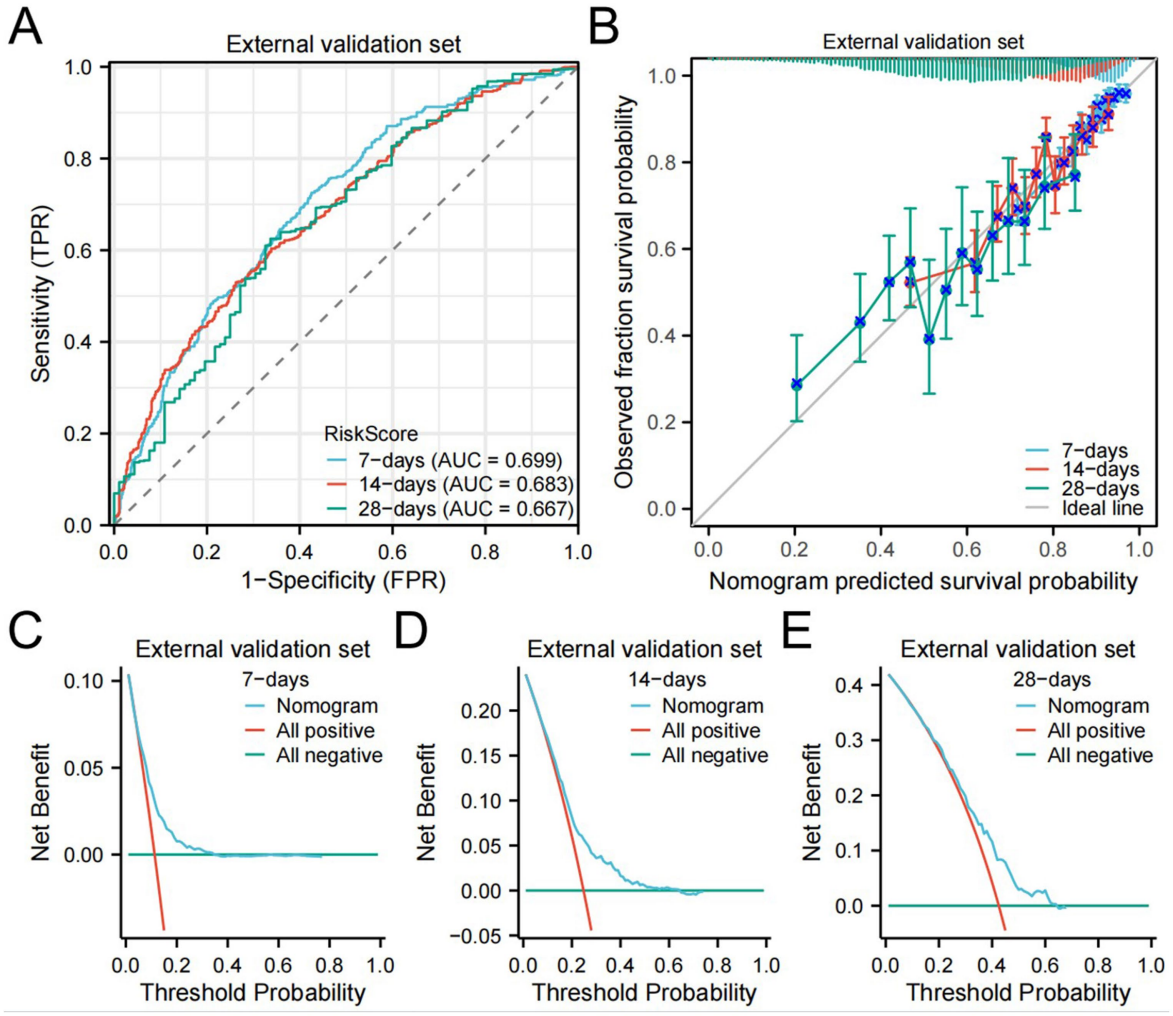
eICU dataset validation results.

The C-index of the external validation set was 0.722 (0.713–0.731). The AUC for 7-day, 14-day, and 28-day mortality risks were 0.699, 0.683, and 0.667, respectively (Figure 6A), indicating that the model exhibited good clinical predictive ability. Calibration curves (Figure 6B) and DCA results (Figures 6C–E) demonstrated good calibration, clinical utility, and net benefit of the model.

## 4 Discussion

Previous studies have shown that COPD patients requiring hospitalization for treatment generally have poorer prognosis, with a mortality rate of approximately 7% (15). The mortality rate of the included COPD patients in this study was 9.1%, which is consistent with epidemiological survey results. This study utilized clinical data of COPD patients extracted from the MIMIC-IV and eICU-CRD databases and employed Cox regression analysis to identify seven independent risk factors associated with the mortality risk of COPD patients. Subsequently, a convenient and effective nomogram model was constructed and validated to predict the short-term survival probability of COPD patients. This model can effectively strengthen the management of COPD patients, as it demonstrates good predictive ability and clinical utility. For instance, if the 7-day survival rate of the patient is high while the 14-day and 28-day survival rates are low, intervention should be carried out as early as possible. It is necessary to closely monitor the changes in the patient’s symptoms and vital signs and enter the ICU for treatment as soon as possible.

A total of 2,036 COPD patients from the MIMIC-IV database were included in this study. The nomogram model developed identified seven predictive factors, including age, weight, APSIII score, ventilator time, potassium, AG, and INR. Among these factors, ventilator time was a protective factor, while the remaining six factors were independent risk factors. Internal and external validation using different data sources demonstrated the robustness and generalizability of our model. Internal validation can evaluate the degree of model fitting, while external validation can assess the generalization ability of the model, reflect the actual clinical application, and enhance the credibility of the model. The C-index and AUC in external validation is lower than internal. The lower performance in external validation may be related to some potential reasons, such as the differences in patient populations, data quality, between the two databases. In patient populations aspects, there may be significant demographic differences between the two databases, such as age, gender, and racial distribution. The severity of the disease, the proportion of comorbidities, and the treatment methods or levels may have differences. For data quality aspect, the MIMIC-IV database is single-center data of the BIDMC which is one of the nation’s preeminent academic medical centers between 2008 and 2019, while the eICU-CRD database is multi-center data of 208 hospitals which are distributed in different region between 2014 and 2015. The data recording methods, inspection and examination equipment, and approaches may vary among different hospitals. BIDMC might have adopted the new technology earlier, while other hospitals might still be using the old ones.

Studies have shown that age is a significant predictor of mortality across various diseases. As individuals age, their quality of life and the physical and functional status of their organs decline, making the older adult more susceptible to various diseases and gradually increasing their mortality rate (16). Epidemiological surveys indicate that the mortality rate among COPD patients increases with age (17). A non-interventional observational study found that the hospitalization and mortality rates of COPD patients over 40 increase with age, possibly due to the increased susceptibility to infections and the risk of lung function deterioration in older adult patients (18, 19).

Multivariate Cox regression analysis revealed an association between body weight and the risk of death in COPD patients. Several studies have confirmed that obesity is an independent risk factor for chronic respiratory diseases, particularly asthma and COPD (20, 21), which aligns with our findings. A Mendelian randomization study indicated that obesity also increases the likelihood of developing chronic obstructive pulmonary disease (Body mass index (BMI): OR = 1.429; waist circumference: OR = 1.591) (22). Obesity results from the excessive accumulation of adipose tissue, which is an active endocrine organ secreting various cytokines and hormones (23). The pathogenesis of COPD is closely related to inflammatory responses. Obese patients often experience persistent low-grade systemic inflammation (24), and increased peripheral blood leukocytes in obese individuals lead to enhanced inflammation and the production of more pro-inflammatory mediators, promoting the progression and exacerbation of COPD (25). Interestingly, some studies suggest that COPD patients often experience weight loss, and a lower BMI is associated with higher hospital mortality rates, as low weight often reflects malnutrition and reduced muscle mass, which may lead to diminished immunity, decreased physical strength, and impaired survival (26), contrary to our findings. However, it is important to note that body weight itself may not be a direct risk or protective factor but is associated with the overall health and nutritional status of the patient.

APSIII score, as part of the APACHE II scoring system is one of the tools used in critical care medicine to assess the severity of a patient’s condition and prognosis, and it is applicable to ICU patients (27). APACHE III covers 12 physiological indicators, including general vital signs, inflammatory markers, and internal environment parameters, providing a more comprehensive quantification of the risk and extent of multi-system damage in patients. APSIII is simpler than the APACHE II score. Therefore, a higher APS score indicates a more unstable physiological state and a more severe condition. The AG is commonly used to evaluate acid–base disturbances and analyze primary metabolic acidosis (28), which is a strong predictor of prognosis in critically ill patients (29). Critically ill COPD patients often develop severe acidemia, leading to myocardial depression, respiratory muscle weakness, increased pro-inflammatory cytokines, and adverse effects on survival prognosis (30). Thus, AG is a risk factor for the prognosis of COPD patients, consistent with our findings.

Potassium ion concentration is an indicator that is often concerned in clinical practice. The Laboratory-Based Intermountain Validated Exacerbation Scores based on laboratory value found that potassium would increase risk of mortality (31), which is consistent with our research results. Moreover, the potassium level of CODP patients with a poor prognosis was significantly lower than that of patients with a good prognosis (32). A retrospective study found that the serum K level of deceased patients with AECODP was significantly lower than that of living patients with AECODP. (33) These are contrary to our findings. This may be related to the fact that CODP patients’ therapeutic drugs (such as *β*₂ receptor agonists, diuretics or glucocorticoids) and complications (such as heart failure, insufficient renal perfusion) may cause abnormal blood potassium levels. When suffering from CODP, carbon dioxide retention can cause respiratory acidosis. At this time, cells exchange and buffer acidic substances through H^+^/K^+^, causing potassium ions to move from the intracellular to the extracellular, which may lead to transient hyperkalemia.

The INR is used to measure the prothrombin time and is crucial for balancing bleeding and coagulation risks (34). COPD patients often exhibit a hypercoagulable state involving changes in various coagulation factors, necessitating anticoagulant therapy, which is monitored through INR levels to assess the effectiveness of the treatment (35). An abnormally high INR may be associated with excessive anticoagulant medication, increasing the risk of bleeding, leading to reduced nutritional status and immunity, and increasing the occurrence of adverse prognostic events, making it a risk factor for patient prognosis. A retrospective clinical study found that the INR in the AECOPD group was significantly higher than in the stable COPD group, indicating that coagulation abnormalities are associated with COPD exacerbations (36). It is noteworthy that while a hypercoagulable state in COPD patients may manifest as a low INR level, the identification of INR as an independent risk factor for COPD prognosis in this study also holds clinical significance.

A retrospective study found that over half (59.3%) of the patients requiring prolonged mechanical ventilation had COPD (37). Another study involving patients on mechanical ventilation for more than 24 h indicated that COPD was one of the most prevalent diseases among the 327 participants (38). As COPD progresses, hypercapnic respiratory failure is one of the causes of death in these patients, with a 1-year mortality rate of 12% (39). Multiple studies have confirmed that long-term non-invasive mechanical ventilation, providing continuous positive airway pressure (CPAP) and supplemental oxygen therapy, can mitigate the negative effects of severe hypercapnia and improve survival rates in COPD patients (40–42).

This study also has some limitations. Firstly, the clinical data we obtained are from different hospitals in different regions, which may introduce some variations in the measurement values. Despite standardization of the data, these differences are inevitable. Secondly, the databases selected for this model are both from hospitals in US and may only reflect some patient information of this country. The use in other countries needs further verification. Thirdly, variables with more than 20% missing values were not included in the study, potentially introducing selection bias and incomplete data, affecting the robustness of the results. Fourthly, as a retrospective cohort study, the nomogram requires further prospective validation before clinical application to increase the reliability of the conclusions. Lastly, the observational nature of the study suggests that unknown confounding factors may influence our results.

## 5 Conclusion

This study successfully developed and validated a simple and effective nomogram model, demonstrating that age, body weight, APS III score, ventilation time, potassium levels, AG, and INR are independent predictors of prognosis in COPD patients. The nomogram model can be used to predict short-term mortality in COPD patients, aiding physicians in identifying high-risk patients, thereby facilitating the development of personalized treatment plans, enhancing patient management, optimizing resource utilization, and reducing mortality rates of COPD patients in clinical practice.

## Data Availability

The original contributions presented in the study are included in the article/supplementary material, further inquiries can be directed to the corresponding authors.
